# The rise of pathogen genomics in Africa

**DOI:** 10.12688/f1000research.147114.1

**Published:** 2024-05-09

**Authors:** Gerald Mboowa, Francis Kakooza, Moses Egesa, Stephen Tukwasibwe, Stephen Kanyerezi, Ivan Sserwadda, Benson R. Kidenya, Jupiter Marina Kabahita, Maria Magdalene Namaganda, Mike Nsubuga, Patricia Nabisubi, Alisen Ayitewala, Grace Kebirungi, Esther Nakafu, Natasha Patience Akwii

**Affiliations:** 1 The African Center of Excellence in Bioinformatics and Data-Intensive Sciences, Infectious Diseases Institute, College of Health Sciences, Makerere University, P.O. Box 22418, Kampala, Uganda; 2 Global Health Security, Infectious Diseases Institute, College of Health Sciences, Makerere University, P.O. Box 22418, Kampala, Uganda; 3 MRC/UVRI & LSHTM Uganda Research Unit, Entebbe, Uganda; 4 Infectious Diseases Research Collaboration, Kampala, Uganda; 5 School of Medicine, Uganda Christian University, Kampala, Uganda; 6 National Health Laboratories and Diagnostics Services, Central Public Health Laboratories, Ministry of Health, P.O Box 7272, Kampala, Uganda; 7 Department of Biochemistry and Molecular Biology, Weill Bugando School of Medicine, Catholic University of Health and Allied Sciences, Mwanza, Tanzania; 8 National Tuberculosis Reference Laboratory/Supranational Reference Laboratory, Plot 106-1062 Butabika Road, Luzira, Uganda; 9 Department of Immunology and Molecular Biology, College of Health Sciences, Makerere University, P.O. Box 77072, Kampala, Uganda; 10 College of Veterinary Medicine, Animal Resources and Bio-security (COVAB), Makerere University, Kampala, Uganda; 11 Department of Plant Sciences, Microbiology and Biotechnology, College of Natural Sciences, Makerere University, Kampala, Uganda

**Keywords:** Africa, Genome sequencing, Pathogen Genomic data, Public health, Data sharing

## Abstract

The routine genomic surveillance of pathogens in diverse geographical settings and equitable data sharing are critical to inform effective infection control and therapeutic development. The coronavirus disease 2019 (COVID-19) pandemic highlighted the importance of routine genomic surveillance of severe acute respiratory syndrome coronavirus 2 (SARS-CoV-2) to detect emerging variants of concern. However, the majority of high-income countries sequenced >0.5% of their COVID-19 cases, unlike low- and middle-income countries. By the end of 2022, many countries around the world had managed to establish capacity for pathogen genomic surveillance. Notably, Beta and Omicron; 2 of the 5 current SARS-CoV-2 variants of concern were first discovered in Africa through an aggressive sequencing campaign led by African scientists. To sustain such infrastructure and expertise beyond this pandemic, other endemic pathogens should leverage this investment. Therefore, countries are establishing multi-pathogen genomic surveillance strategies. Here we provide a catalog of the current landscape of sequenced and publicly shared pathogens in different countries in Africa. Drawing upon our collective knowledge and expertise, we review the ever-evolving challenges and propose innovative recommendations.

## Introduction

The global genomic surveillance strategy for pathogens with pandemic and epidemic potential, 2022–2032 was established by the World Health Organisation (WHO) to help countries develop their national genomic surveillance strategy for priority pathogens. ^1^ The regular collection and sharing of such data are also fundamental for monitoring and effectively responding to outbreaks and for tracking antimicrobial resistance (AMR) to inform decision-making. Furthermore, WHO emphasizes that sharing of such data is critical for preventing, detecting, and timely responding to epidemics and pandemics at all levels, and is in the interest of all Member States. ^2^

Africa remains increasingly prone to infectious disease threats, recording at least 140 disease outbreaks annually ^3^^,^^4^ besides the high burden of drug resistance that has been estimated to have caused at least 1.05 million deaths in 2019. ^5^ This calls for an urgent need to build and sustain capacity for near real-time outbreak detection, characterization, and routine pathogen genomic surveillance. Coronavirus disease (COVID-19) showed that most of the public health institutions in Africa if adequately capacitated and facilitated with high-throughput sequencing platforms, reagents, and data analytics infrastructure, can perform rapid pathogen detection, characterization, surveillance, and equitable data sharing. As of January 4, 2024, over 170,000 Severe acute respiratory syndrome coronavirus 2 (SARS-CoV-2) genomes from 53 out of 55 countries in Africa have been sequenced and shared via GISAID (the Global Initiative on Sharing All Influenza Data). ^6^ An important lesson to learn is that we need to facilitate national laboratories and local scientists to undertake routine pathogen sequencing activities within their countries while collaborating with the rest of the globe. The continent still has several pathogens of epidemic and pandemic potential that require near real-time tracking and in response, the Africa Centres for Disease Control and Prevention (Africa CDC) generated a list of priority pathogens for effective emergency preparedness and response. ^7^

## Pathogen sequencing in Africa

Next-generation sequencing (NGS) is improving how disease outbreaks are accurately detected and investigated at an unparalleled magnitude. Most public health and research laboratories in many countries including Africa have at least one of the common NGS platforms (see Table 1). Different variables affect the choice of NGS platforms to be acquired by an institution such as the cost of the instrument, availability of after-sale service, access and cost of reagents, sequencing run time, throughput, accuracy, strategies for using NGS (targeted/panel, whole-exome, and whole-genome), depth of sequencing coverage, length of sequencing reads, single-end versus paired-end sequencing, genome size of an organism to be sequenced.

**Table 1.** Key specifications of common NGS throughput platforms.

Throughput sequencing categories/Data output range (Gb: gigabyte and Tb: terabyte)	Common equipment/ Maximum Read Length (base pairs) /Maximum Raw data Output (Gb: gigabyte and Tb: terabyte)	Key applications
Low throughput (1–100 Gb)	iSeq 100-2x150 bp - 1.2 Gb MiniSeq - 2x150 bp - 7.5 Gb DNBSEQ-E25-2x150 bp - 7.5 Gb Ion Torrent: Proton/PGM - 400 bp -10 Gb PacBio RS II - 60 kb - 10 Gb MiSeq - 2x300 bp - 15 Gb MinION - 100 kb - 50 Gb DNBSEQ-G99-2x300 bp - 96 Gb	Targeted panel sequencing Pathogen sequencing (microbes and viruses) 16S metagenomic sequencing
Medium throughput (100–1000 Gb)	NextSeq - 2x150 bp - 120 Gb DNBSEQ-G50RS - 2x150 bp - 150 Gb GridION - 100 kb - 250 Gb PacBio Sequel II/IIe - 60 kb - 500 Gb	Small whole-genome sequencing Targeted panel sequencing (amplicon and gene panel) Gene expression profiling with mRNA-Seq miRNA and Small RNA analysis
High throughput (1000–10 000 Gb)	DNBSEQ-G400RS - 2 x 300 bp - 1.4 Tb HiSeq - 2 x 150 bp - 1.5 Tb DNBSEQ-T7-2 x 150 bp - 7 Tb	Whole-genome sequencing Whole-exome sequencing Transcriptome sequencing Shotgun metagenomics
Ultra-high throughput (>10 Tb)	PromethION - 270 kb - 13.3 Tb NovaSeq - 2x250 bp - 16 Tb DNBSEQ-T20×2RS - 2x150 bp - 72 Tb DNBSEQ-T10×4RS - 2x150 bp - 76.8 Tb	Ultra-high-depth whole-genome sequencing of large genomes (Human, plant and animal) Whole-exome sequencing Whole-transcriptome sequencing Methylation sequencing Shotgun metagenomics

**Figure 1.**
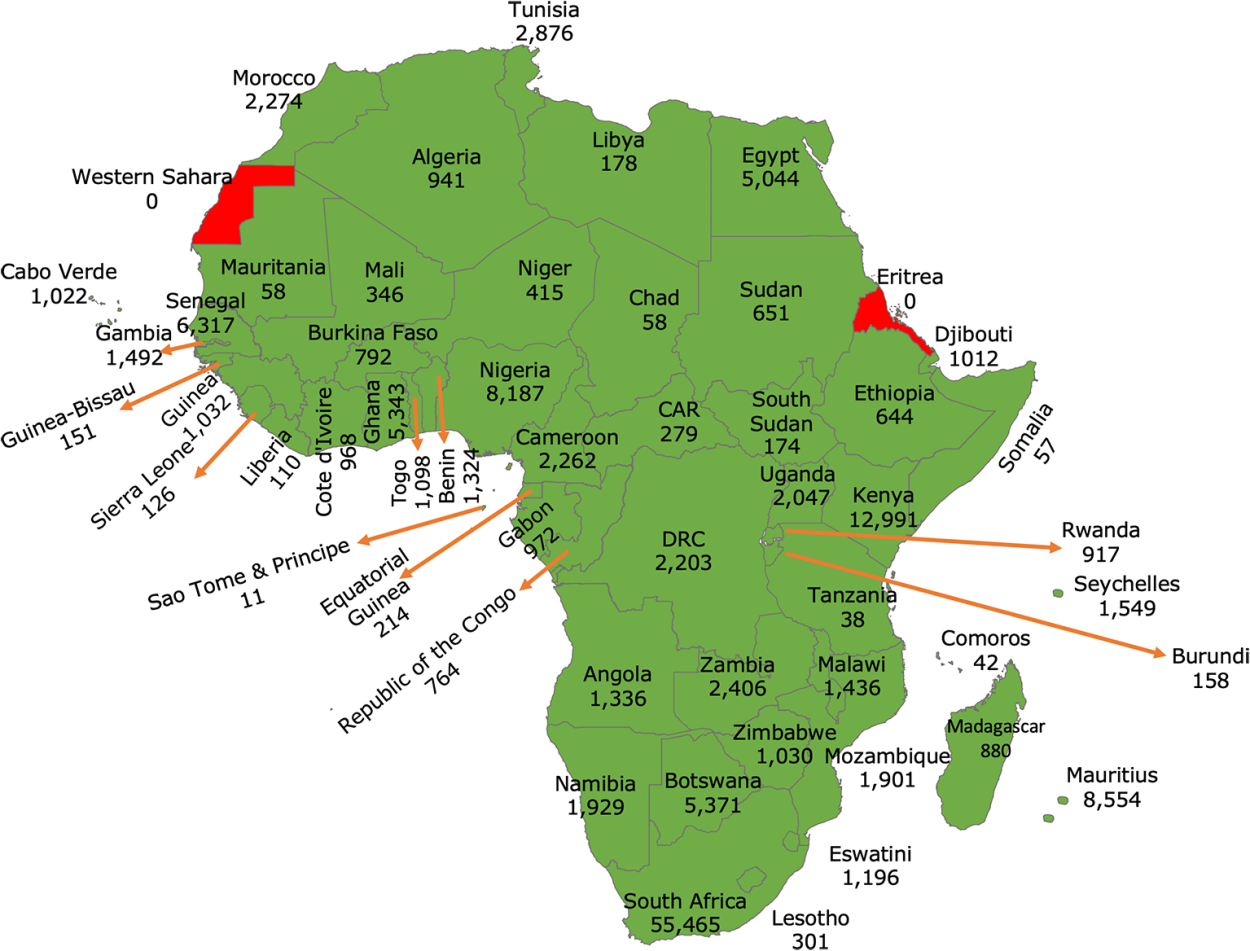
Map of Africa showing the number of SARS-CoV-2 genomes sequenced and shared via GISAID per country. (GISAID data accessed on January 4, 2024). A total of 53/55 African Union Member States sequenced and shared their SARS-CoV-2 genomes since the start of the pandemic. At least 96% sequenced by Hubs based in Africa. ^9^

COVID-19 flung pathogen genome sequencing to the vanguard of accurate disease outbreak detection, characterization, surveillance of emerging variants, and real-time data sharing. But in the first two years of the pandemic, 78% of high-income settings sequenced >0.5% of their diagnosed COVID-19 cases, compared to 42% of low- and middle-income countries. ^8^ Following this inequality, high-income countries were encouraged to support low- and middle-income countries to improve their local sequencing capacity ^8^ to ensure an effective global response to the pandemic. Furthermore, a study revealed that sequencing 0.5% of the cases with a turnaround time of less than 21 days could provide a benchmark for SARS-CoV-2 routine genomic surveillance to detect emerging variants. ^8^ In Africa, our findings suggest that many countries have built local capacity for pathogen genomics (see Figure 1). This was possible in part because, in the early phase of the pandemic, COVID-19-related travel restrictions forced countries to consider building local capacity for molecular testing and genomic surveillance of SARS-CoV-2 variants other than relying on shipping samples to other countries. By January 4, 2024, the continent had sequenced at least 1.3% of the reported cases (see Table 2) and ranked fourth.

**Table 2.** Shows proportions of SARS-CoV-2 cases sequenced by each continent. Reported number of COVID-19 cases * and SARS-CoV-2 sequence data shared via the Global Initiative on Sharing All Influenza Data (GISAID) ^#^ by each continent. Data accessed on January 4, 2024.

Continent	COVID-19 cases	Sequenced SARS-CoV-2 genomes in GISAID	Percentage of COVID cases sequenced & shared
North America	130,243,977	5,781,894	4.40%
Europe	252,605,493	8,027,917	3.20%
Oceania	14,722,723	283,139	1.90%
Africa	12,857,043	171,881	1.30%
Asia	221,152,538	1,701,264	0.80%
South America	69,454,296	398,332	0.60%

* ( https://www.worldometers.info/coronavirus/?utm_campaign=homeAdUOA?Si%23countries).

^#^ ( https://gisaid.org/).

**Figure 2.**
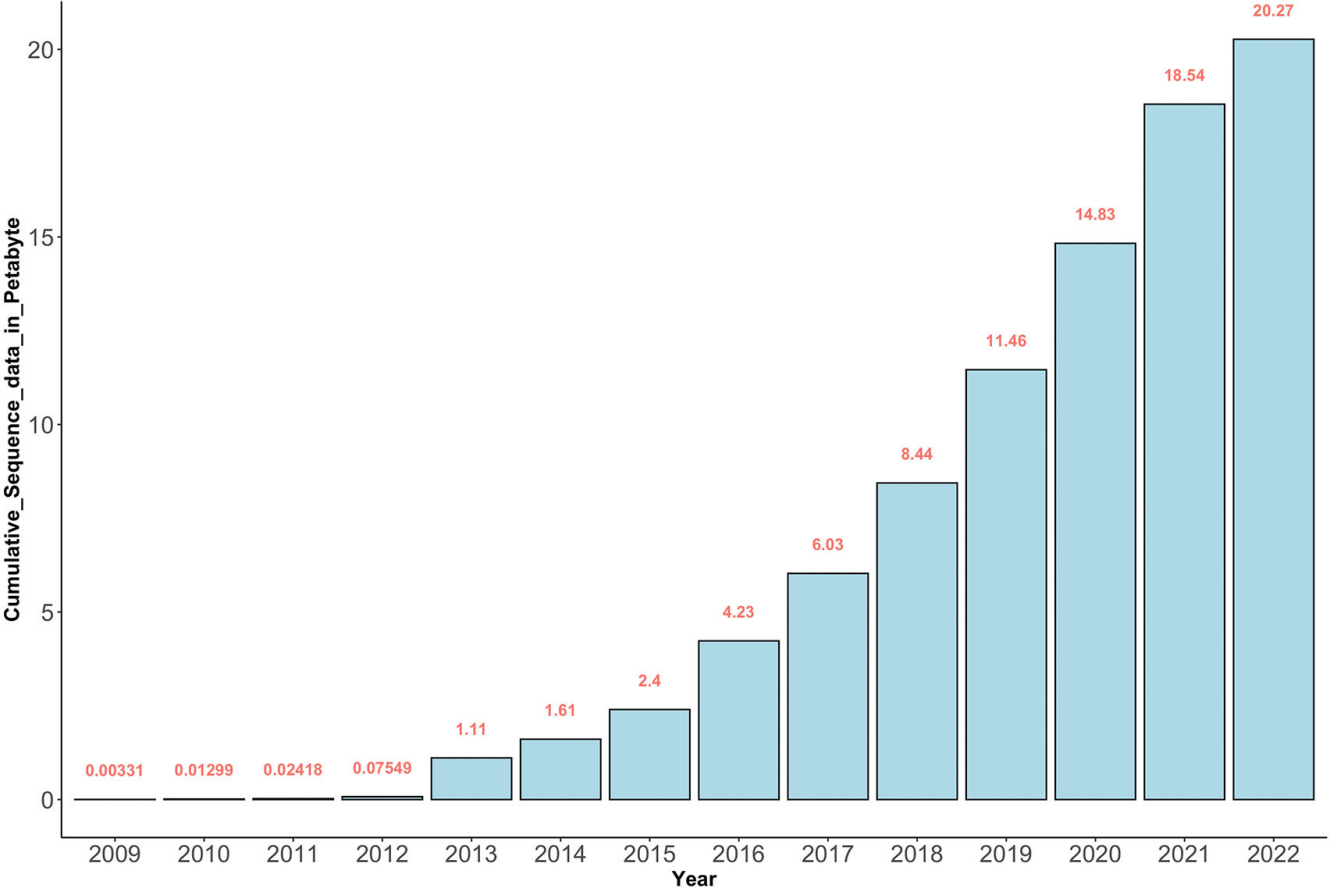
The cumulative amount of genomic sequence data in Petabytes deposited in SRA since 2009. Between 2012 and 2022 (~10 years), there were at least 20.2 Petabytes of sequence data deposited in SRA.

Generally, the reduced costs of genome sequencing have led to the generation of large volumes of genomic sequence data globally as seen from the shared data in the Sequence Read Archive (SRA) for NGS data at the National Center for Biotechnology Information (NCBI) (see Figure 2). The SRA was established as part of the International Nucleotide Sequence Database Collaboration (INSDC) in 2009. ^10^

It should be noted that more genomic data is being generated than the capacity to analyze and meaningfully interpret it. ^11^^,^^12^ This data if well utilized holds clues to improve health. This makes FAIRness (findability, accessibility, interoperability, and reusability) data sharing ^13^ a critical component in this ecosystem so that scientists in well-resourced settings can collaboratively and equitably derive valuable insights from this data.

## Data sharing of pathogen genomic data

COVID-19 propelled two important components in effective infectious disease response; (i) near-real-time pathogen sequencing and (ii) unprecedented collaborative scale in pathogen data sharing with over 16.4 million SARS-CoV-2 genomes already publicly shared via GISAID from all over the world by January 4, 2024.

Since most of the SARS-CoV-2 sequence data was shared via GISAID, we searched the SRA/NCBI database ( https://www.ncbi.nlm.nih.gov/sra/) up until January 4, 2024, for publicly accessible sequence data for other pathogens from different countries in Africa. The search terms used were, “Pathogen [All Fields] AND Country [All Fields]”. The retrieved data included different pathogens sequenced from each country filtered by microbial taxon, BioProject, genomic library layout, NGS platform, volume of data, and submitting/sequencing institution (see Extended data: Table 3 ^14^).

In Africa before the COVID-19 pandemic, sequencing was largely a sophisticated endeavor undertaken by individuals and institutions based in high-income settings in collaboration with African institutions (see Extended data: Table 3 ^14^). The local institutions largely participated in sample collection and processing for shipping to institutions in well-resourced countries. This implied that the objectives of these sequencing activities were also addressing the research aims of institutions in high-income settings. The pandemic brought about a paradigm shift that allowed local public health institutions to mobilize resources to embrace the genomic revolution in public health. This has been largely achieved through the Africa Pathogen Genomics Initiative (Africa PGI), ^4^ which has equipped, trained, and offered technical support for African Union (AU) Member States to build sustainable genomics and bioinformatics capacity. Furthermore, the AU Commission and Africa CDC have called on governments, multi-lateral organizations, philanthropies, the private sector, and civil society organizations to support the full implementation of Africa’s New Public Health Order to drive global health security. ^15^ In addition to this, it is imperative to promote equitable data sharing for pathogens of epidemic and pandemic potential in Africa while discouraging post-outbreak sanctions on countries that share data.

## Major emerging challenges

The ever-growing volume of genomic sequence data coming out of laboratories in Africa has amplified interest in genomics and bioinformatics applications in public health. ^16^ However, majority of the workforce has limited experience in these fields largely due to the limited number of training institutions that can offer the training. It should be noted that meaningful interpretation of genomic data generated from disease outbreaks requires adequate training and collaborative efforts (within and outside the continent). Sequencing facilities have varying levels of expertise in sequencing different organisms such as viruses, bacteria, fungi, and parasites as well as different sequencing technologies.

There is an urgent need for public health institutions in Africa to recruit and retain competent personnel dedicated to performing bioinformatics analyses that can support evidence-informed public health decision-making. In some cases, it has been complicated to establish such job positions within institutional structures. As such resorting to training the available personnel in this field is the most reasonable option however, this training requires long periods of learning, mentorship, and hands-on practice. ^17^

Limited local data analytics infrastructure is another critical emerging challenge. With many institutions having access to local NGS sequencing platforms that generate large volumes of raw sequence data that require memory-intensive analytic compute and storage, there is an ever-growing need to provide access to local high-performance computing (HPC) facilities. Setting up HPC facilities requires a lot of money and creates new challenges such as increasing electricity consumption and a need to recruit a competent HPC systems administrator. Furthermore, utilization of cloud-based resources for bioinformatics analyses also requires access to stable fast internet that may be unavailable in some countries.

The need to invest in biobanking and biorepository services in Africa. These facilities archive and distribute well-characterized biospecimens for research to support the development of disease diagnostics and therapeutics. ^18^ Genomics activities rely on access to samples in these facilities. Many public health laboratories faced the challenge of storing thousands of samples during 2014-2016 Ebola outbreak in West Africa and the COVID-19 outbreak and realized a need to expand their biobanking capacities.

Challenges of timely access to NGS reagents and supply chain. Travel restrictions during COVID-19 highlighted the need to improve the supply chain of NGS reagents in countries that lacked or had limited access to channel partners. Routine genomic surveillance requires reliable access to affordable NGS reagents and after-sales service. It is estimated that the cost per pathogen sequence ranges from US $20–$200, with poor countries paying up to 10-fold more than high-income countries. ^19^

Pathogen genomics in public health in Africa requires continuous engagement among different stakeholders including NGS platform manufacturers/distributors, program/project funders, sequencing facilities, research/academic institutions, the national ministries of health, and policymakers. Each of these has a unique contribution, therefore planning and supporting multi-pathogen activities is a multi-sectoral endeavor that requires continued engagement to ensure an impactful genomic ecosystem. For example, the prohibitive cost of using onboard NGS instrument data analysis software licenses requires engagement with platform manufacturers. Cloud-based computing is an enthralling emerging infrastructure that offers an alternative infrastructure for solving traditional bioinformatics challenges in NGS data analytics and visualization. Cloud computing service models are classified into three types: Platform as a Service (PaaS), Software as a Service (SaaS), and Infrastructure as a Service (IaaS). ^20^ The platform is relatively easier to use than the Linux command-line-based environment however it requires a pay-as-you-go subscription that charges based on usage.

Recent years have revealed an emerging dimension of the impact of climate change on the evolution of pathogens in Africa. ^21^^,^^22^ For example, climate change and extreme weather conditions have triggered increased cholera outbreaks in Africa. ^23^ Research on modeling climate change and pathogen genomic data has failed to be socially inclusive, geographically balanced, and broad in terms of the disease systems studied, limiting our capacities to better understand the actual contribution of climate change on disease outbreaks. ^22^ Therefore, integrating climate change data in pathogen genomics is important, and leveraging this expertise through collaboration with institutions that have established capacity.

## Beyond the post-COVID-19 genomics investment in Africa and way forward

COVID-19 allowed most of the countries in Africa to build local capacity for pathogen genome sequencing. It is important to keep the momentum by leveraging this investment to fight the continent’s endemic health challenges including Malaria, Cholera, HIV/AIDS, Tuberculosis, Vaccine-preventable diseases, Viral hemorrhagic fevers (VHFs) and the growing threat of antimicrobial resistance. Therefore, as recommended by the WHO, ^1^ countries are establishing multi-pathogen genomic surveillance strategies that will ensure they can rapidly detect and precisely characterize pathogens, but this also requires stable funding from national governments and other funding agencies such as the Pandemic Fund.

**Figure 3.**
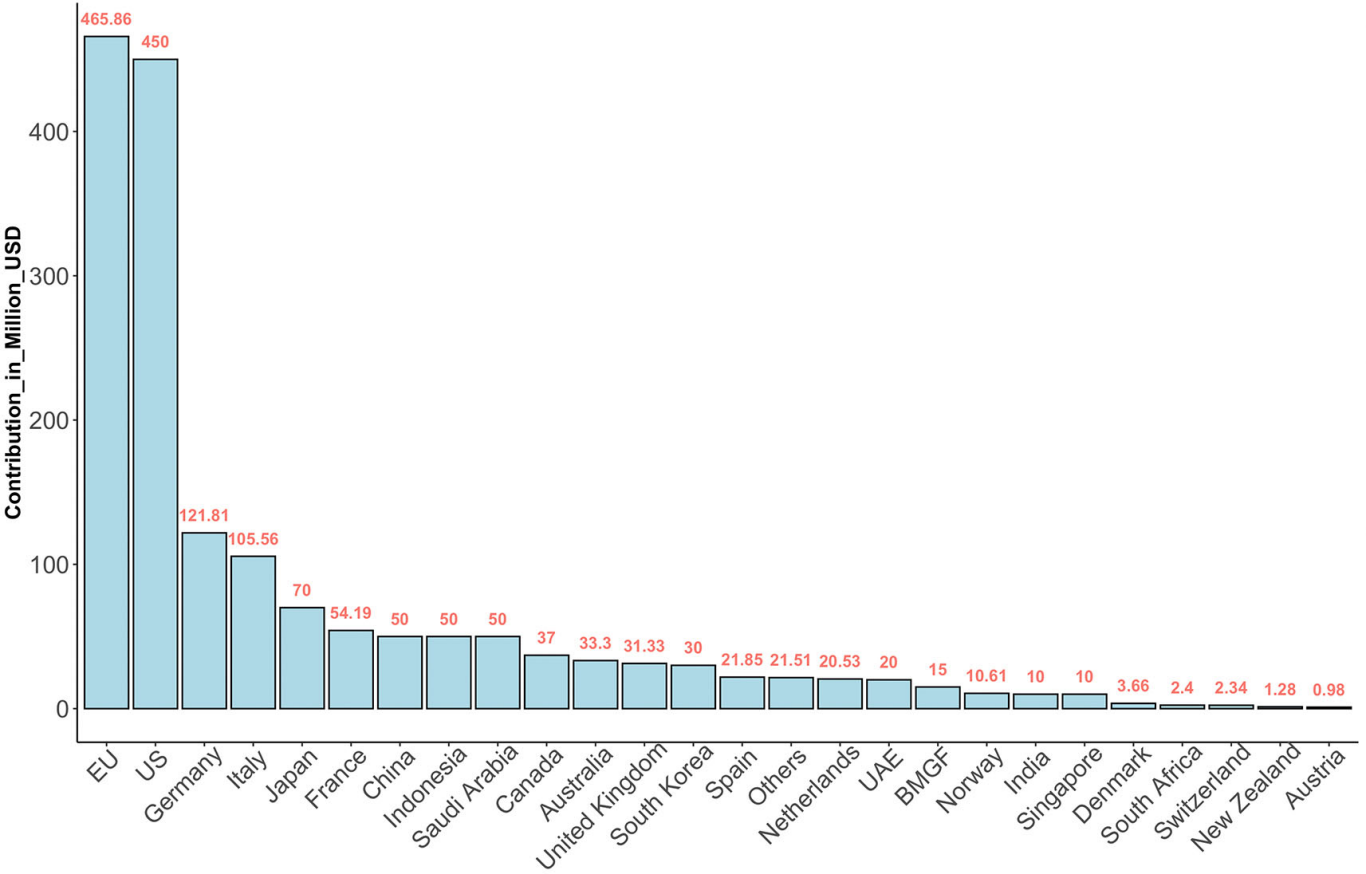
Funds contributed to the Pandemic Fund. The Fund aims to help countries build resilience to future pandemics. The data was accessed on January 4, 2024 ( https://fiftrustee.worldbank.org/en/about/unit/dfi/fiftrustee/fund-detail/pppr).

The global economic cost of the COVID-19 pandemic was estimated at USD $16 trillion. ^24^ Much as countries now appreciate the benefits of routine pathogen surveillance, many in Africa require technical and financial support. The devastating human, economic, and social cost of COVID-19 highlighted the urgent need for well-coordinated action to establish stronger health systems and mobilize additional resources for pandemic prevention, preparedness, response, and serve as a platform for advocacy. The WHO coordinated efforts through the newly established Pandemic Fund, which is a collaborative partnership among donor governments, co-investor countries, foundations, civil society organizations, and international agencies providing a dedicated stream of long-term funding (see Figure 3) for critical pandemic prevention, preparedness, and response (PPR) for future pandemics. The Pandemic Fund is set to finance critical investments to strengthen pandemic prevention, preparedness, and response capacities at national, regional, and global levels, with a focus on low- and middle-income countries. ^25^

In 2023, projects that were financed prioritized strengthening comprehensive outbreak surveillance and early warning, laboratory systems, and human resources/public health workforce capacity. The Pandemic Fund is set to allocate financing where investments are most urgently needed to bolster PPR for future pandemics, addressing key capacity gaps at national, regional, and global levels. Overall, the Fund’s aims include: (i) supporting surveillance systems that enable timely tracking and reporting of outbreaks, (ii) creating faster and more accurate data sharing and (iii) building better laboratories so that they better assist partner countries to rapidly detect and effectively respond to infectious disease outbreaks.

Beyond analysis, reporting, and data sharing of pathogen genomic data, the continent also needs to utilize the genomics resource to improve local research and development (R&D) capacity as well as support local therapeutics and affordable diagnostic manufacturing. The Partnerships for African Vaccine Manufacturing (PAVM) has a goal to enable the African vaccine manufacturing industry to develop, produce, and supply over 60 percent of the total vaccine doses required to fight endemic diseases on the continent by 2040. ^26^ This offers an exceptional opportunity for enhancing local vaccine training. Rwanda is a pioneering country, hosting the first mRNA vaccine manufacturing facility in Africa, and soon, Senegal, South Africa, and Kenya will be the other African countries to have mRNA vaccine manufacturing facilities on the continent. ^27^

## Conclusions

Timely and accurate analysis of pathogen genomes is crucial for monitoring the molecular evolution and dissemination of pathogens, improving diagnostic molecular tests and vaccines, and guiding efficient public health interventions. Therefore, there is an urgent need to sustain gains in local pathogen genomics in Africa while strengthening data analytics infrastructure and an equitable data sharing ecosystem.

## Data Availability

No data are associated with this article. Zenodo: Extended data for ‘The rise of pathogen genomics in Africa’, https://doi.org/10.6084/m9.figshare.25574040.v1. ^14^ This project contains following extended data: Table 3. The different pathogens that have been sequenced and publicly shared via SRA:NCBI from different countries in Africa (at least 12 Terabytes of sequence data).xlsx Data are available under the terms of the Creative Commons Attribution 4.0 International license (CC-BY 4.0)
