# Supplementary material for: The rise of pathogen genomics in Africa
Source: F1000Res. 2024 Sep 4;13:468. Originally published 2024 May 9. [Version 2] doi: 10.12688/f1000research.147114.2 (PMC11512136; doi:10.12688/f1000research.147114.2)
Supplement: Peer review history

# Reviewer report: Reviewer response for version 2

**DOI:** 10.5256/f1000research.170999.r330025

**Published:** 2024-10-28

**Authors:** Onikepe Folarin

**Affiliations:** 1 Redeemer's University, Ede, Osun, Nigeria

The authors review is the genomics efforts particularly for disease diagnosis in Africa. The authors made reference to the COVID-19 pandemic and subsequent efforts in Africa to develop capacity in genomics in order to effectively control and manage the menace of infectious diseases which is a major public health challenge in Africa. In addition, the authors touched on challenges facing genomics research and capacity in Africa which include capital, human capacity, supply chain etc.

 The review showed the fact that Africa is making efforts to get on the fast moving genomics train however with the challenges stated it might take a while to get Africa fully on board.

 One of the the statement i did not see in the review is the fact that while Africa is able to sequence lots of the COVID-19 cases, this was done by the efforts of some genomics centers/Institutions  thus the more need for the capacity (both human and infrastructure). 

 While training is emphasized in the recommendations, other contributions to be considered include African government buying in to genomics and genomics research. The same way Africa decided to take charge of controlling COVID-19 during the pandemic, the African government need to support and invest in genomics in Africa. This by allowing the establishment of biotechnology in Africa to take care of challenges of supply chain as well as support genomics research in the country. Another suggested recommendation is the need for Africans to collaborate in genomics research which also was one of the issue that played out during COVID-19 where African scientists collaborated to share methodologies and facilities. 

 The manuscript is well written and content is adequate for indexing considering other points raised.

Is the topic of the opinion article discussed accurately in the context of the current literature?

Yes

Are arguments sufficiently supported by evidence from the published literature?

Yes

Are all factual statements correct and adequately supported by citations?

Yes

Are the conclusions drawn balanced and justified on the basis of the presented arguments?

Yes

Reviewer Expertise:

Infectious diseases; Genomics

I confirm that I have read this submission and believe that I have an appropriate level of expertise to confirm that it is of an acceptable scientific standard.

# Reviewer report: Reviewer response for version 2

**DOI:** 10.5256/f1000research.170999.r330026

**Published:** 2024-10-25

**Authors:** Gabriel E. Wagner

**Affiliations:** 1 Medical University of Graz, Graz, Austria

The opinion article, *"The rise of pathogen genomics in Africa"* by Gerald Mboowa and colleagues provides an detailed overview of the sequencing capacities and the efforts taken to further establish molecular pathogen surveillance in Africa during the COVID-19 pandemic.

 The article provides a brief introduction to genomic pathogen surveillance and why it is so important to build capacity for the necessary sequencing equipment and bioinformatics. Mboowa and colleagues show very nicely what has happened in this area during the COVID-19 pandemic and how this considerable progress has led to important contributions in and from Africa. There is no question that these competences should continue to be maintained and expanded, as this benefits not only Africa but all of us as a global scientific community - as very well demonstrated on the example of SARS-CoV-2 in the publication. One of the major factors in this area, as the article argues, will definitely be to offer comprehensive bioinformatics expertise and training - the initiatives mentioned in the article can serve as a starting point for the interested reader. The article is definitely an important contribution and provides a great overview of progress and problems in pathogen surveillance in Africa, although a few small changes could further improve readability and impact.

- In the abstract: „ However, the majority of high-income countries sequenced >0.5% of their COVID-19 cases, unlike low- and middle-income countries.”. I would add a number here, what is the difference to low and middle income countries.
  Of note, also for the paragraph in the text: I am not convinced that the percentage of sequenced COVID cases is a good estimator. It depends on which COVID tests were used and how extensively they are used. Furthermore, one could argue that waste water surveillance also provides a very good overview and is very cost-efficient.
- In my view, the publication would benefit from including examples of what genomic surveillance has already achieved in Africa. It is very well elaborated what capacities exist and what needs to be established on a larger scale, but what is the benefit when all goals are achieved? This would emphasize the importance of the topic in my opinion, e.g. extend on the link between NGS and vaccine adaptations.
- In general, the article is very "COVID-heavy". Based on the title, I would have expected a somewhat broader scope here. Could other initiatives for other pathogens perhaps be mentioned here?
  In this context. The authors mention: ‘The continent still has several pathogens of epidemic and pandemic potential that require near real-time tracking’, a brief overview of which pathogens are meant or what ‘near real-time tracking’ is used for would be useful here. It is not entirely clear which time period is meant.
- Table 1 is pretty big and it’s certainly important for networking purposes so it should not be excluded. However, I might suggest to move the country list with the specific NGS equipment to the supplementary.
- The key applications in Table 1 are a bit misleading in my opinion, e.g. one could easily run 12-20 bacterial WGS samples on a minion (or even more on a MiSq). It all depends on the number of samples and the amount of data required for the analysis. It may be better to have two tables here. One that gives information about the devices and their output and a second one with applications and their requirements? Depending on the number of samples one can then look for the right device here.

- The information on the MinION is not consistent, in Table 1 an output of 50Gb is specified, on page 9, however, 15-20Gb are stated. Further note: The read length for ONT devices is limited mostly by the DNA Prep, the maximum read length is questionable here (especially since different maximum read lengths were specified for different ONT devices). The longest read ever was over 4Mb until recently.
- The search term “Pathogen [All Fields] AND Country [All Fields]” on the SRA webpage ( https://www.ncbi.nlm.nih.gov/sra/) as describe on page 9  delivers pathogens from all countries, not just Africa?
- The paragraphs under “Major emerging challenges” are a bit redundant and I’d generally suggest to shorten them a bit. In my view, the key messages are sometimes a little lost. The authors could further comment critically here that with cloud platforms, to a certain extent, one also gives the data out of ones hands and makes oneself dependent on certain providers again.
- The paragraph „ Challenges of integrating ethics and law in international collaborations” might also be shorten a bit.

In my view, the points above would emphasize the intentions of the article a little more and increase the flow of reading. Nevertheless, it is already an excellent and above all important article.

Is the topic of the opinion article discussed accurately in the context of the current literature?

Yes

Are arguments sufficiently supported by evidence from the published literature?

Yes

Are all factual statements correct and adequately supported by citations?

Yes

Are the conclusions drawn balanced and justified on the basis of the presented arguments?

Partly

Reviewer Expertise:

Molecular microbiology

I confirm that I have read this submission and believe that I have an appropriate level of expertise to confirm that it is of an acceptable scientific standard, however I have significant reservations, as outlined above.

# Reviewer report: Reviewer response for version 2

**DOI:** 10.5256/f1000research.170999.r320742

**Published:** 2024-09-25

**Authors:** Erik Bongcam-Rudloff

**Affiliations:** 1 Department of Animal Biosciences, Swedish University of Agricultural Sciences, Uppsala, Sweden

The revisions made by the authors are satisfactory and align well with the recommendations provided during the initial review.

Is the topic of the opinion article discussed accurately in the context of the current literature?

Yes

Are arguments sufficiently supported by evidence from the published literature?

Partly

Are all factual statements correct and adequately supported by citations?

Yes

Are the conclusions drawn balanced and justified on the basis of the presented arguments?

Partly

Reviewer Expertise:

Bioinformatics, genomics, cell biology, molecular biology

I confirm that I have read this submission and believe that I have an appropriate level of expertise to confirm that it is of an acceptable scientific standard.

# Reviewer report: Reviewer response for version 1

**DOI:** 10.5256/f1000research.161264.r276910

**Published:** 2024-07-03

**Authors:** Ana S Gonzalez-Reiche

**Affiliations:** 1 Icahn School of Medicine at Mount Sinai, New York, USA

The article by Mboowa and collaborators describes the resources available in Africa for genomic pathogen surveillance and how these were built, strengthened, or expanded across the continent due to the COVID-19 pandemic. This important topic deserves attention as it prepares us to respond to emerging infectious diseases globally. Most importantly, it underscores the availability of local capacity and the potential to reduce the gap in research led by African scientists through their perspective. 

 The article would benefit from highlighting the data generated and the resources available in Africa—for example, Table 1. Rather than listing common NGS platforms, list those available in Africa alone. The table header makes it unclear whether this table is already specific to Africa. 

 Similarly, in Figure 2, the authors could include an insert plot showing the distribution of SRA data for Africa only. 

 Another essential information would be how these resources are distributed across academic, healthcare, government institutions, and the public and private sectors. This could help lay out a strategic plan to synergize better current efforts to build a sustainable long-term surveillance program. If any of this information is available to the authors, it should be included. 

 The section " **Major emerging challenges"** could more effectively state the needs if concrete estimates were included. For example, in the third paragraph of this section, the authors could provide an estimate of resources (e.g. computing capacity) and money (e.g. estimated cost per processor/computing node) that would be needed to sustain a genomic surveillance lab sequencing X samples per year. This would be a more practical comparison highlighting the increased cost of sequencing in low-income countries where the supply chains for sequencing reagents aren’t as robust as those available to high-income countries. Using one or two of the author's institutions as examples should suffice.

 Similarly, in paragraph 6, it seems to this reviewer that investing in building local capacity for bioinformatic analysis will probably pay off in the long run compared to investing in license-based software. This is important as it will highlight the need to establish a critical mass of bioinformatics expertise, including bioinformatic training at universities, etc. (or support for existing training options). A brief cost analysis could also help to support this.

 In the section “ **Beyond the Post-COVID-19 genomics…**” The Pandemic Fund initiative should be introduced at its first mention. The authors introduce the Pandemic Fund in the second paragraph, but they first mention it at the end of the first paragraph. Please consider reorganizing this section to address this.

Is the topic of the opinion article discussed accurately in the context of the current literature?

Yes

Are arguments sufficiently supported by evidence from the published literature?

Partly

Are all factual statements correct and adequately supported by citations?

Yes

Are the conclusions drawn balanced and justified on the basis of the presented arguments?

Partly

Reviewer Expertise:

Genomic Pathogen Surveillance, SARS-CoV-2, Respiratory Viruses, Viral Sequencing,  Bioinformatics

I confirm that I have read this submission and believe that I have an appropriate level of expertise to confirm that it is of an acceptable scientific standard, however I have significant reservations, as outlined above.

# Reviewer report: Reviewer response for version 1

**DOI:** 10.5256/f1000research.161264.r278094

**Published:** 2024-06-11

**Authors:** Erik Bongcam-Rudloff

**Affiliations:** 1 Department of Animal Biosciences, Swedish University of Agricultural Sciences, Uppsala, Sweden

The authors discuss an important issue regarding the significant threat that new pathogens pose to humanity. Specifically, the article addresses the situation in the African continent and the need for a Pan-African developed early surveillance system.

 The authors accurately describe the use of NGS technologies and the necessity of high-throughput computing systems and high-speed internet to share and analyse common sequence data. 

 However, the article lacks a more detailed discussion about the need for a critical mass of researchers with the bioinformatics competence to perform these tasks at a pan-African level. The article should address and mention some of the existing education initiatives (e.g., AI4D Africa, H3Abionet, EANBiT, University programmes, etc.) and the imperative need to educate more African scientists in using Artificial Intelligence, HPC systems, NGS sequencing, and bioinformatics analysis.

 International collaboration relies on the integration of ethics and law across national legal systems. The article would benefit from discussing the challenges and problems that can arise when considering ethical and legal issues in such collaborations.

Is the topic of the opinion article discussed accurately in the context of the current literature?

Yes

Are arguments sufficiently supported by evidence from the published literature?

Partly

Are all factual statements correct and adequately supported by citations?

Yes

Are the conclusions drawn balanced and justified on the basis of the presented arguments?

Partly

Reviewer Expertise:

Medical Sciences, Bioinformatics, Biobanking, Exposome research, Metagenomics, Genomics, HPC

I confirm that I have read this submission and believe that I have an appropriate level of expertise to confirm that it is of an acceptable scientific standard, however I have significant reservations, as outlined above.

## References

[1] : Regulation of genomic and biobanking research in Africa: a content analysis of ethics guidelines, policies and procedures from 22 African countries. *BMC Med Ethics* .2017;18(1) : 10.1186/s12910-016-0165-6 8 10.1186/s12910-016-0165-6 28153006 PMC5289015

[2] : Legal & ethical compliance when sharing biospecimen. *Brief Funct Genomics* .2018;17(1) : 10.1093/bfgp/elx008 1-7 10.1093/bfgp/elx008 28460118 PMC5859993
